# Theoretical models: necessary reflections

**DOI:** 10.1590/1980-549720230038.2

**Published:** 2023-09-18

**Authors:** Breno Augusto Bormann de Souza, Érika Fernandes Tritany, Gustavo Alonso Cabrera Arana, Cláudio José Struchiner

**Affiliations:** I Fundação Oswaldo Cruz, Escola Nacional de Saúde Pública, Departamento de Epidemiologia – Rio de Janeiro (RJ), Brasil.; II Universidade de Pernambuco – Recife (PE), Brasil.; III Universidade Federal do Rio de Janeiro – Macaé (RJ), Brasil.; IV Universidade Federal do Rio Grande do Norte – Natal (RN), Brasil.; V Faculdade de Saúde Pública da Universidade de Antioquia – Medelín, Colômbia.; VI Fundação Getulio Vargas – Rio de Janeiro (RJ), Brasil.; VII Universidade Estadual do Rio de Janeiro – Rio de Janeiro (RJ), Brasil.

**Keywords:** Models, theoretical, Grounded theory, Causality, Research report, Publications, Modelos teóricos, Teoria fundamentada, Causalidade, Relatório de pesquisa, Publicações

## Abstract

**Objective:**

To present theoretical-methodological reflections on the elaboration, types, and functions of theoretical models as well as their conceptual and analytic frameworks.

**Methods:**

This is an essay, whose material collection was carried out in a non-systematic way, by electing studies exclusively based on the line of argument and reflection that the authors intend to submit to appreciation and public debate.

**Results:**

We present reflections on the types and functions of theoretical models, theoretical foundations in research, and reflections on the importance of theoretical models for public health research and their relation with the process of elaboration, development, and reporting in scientific studies. In addition, we describe types of theoretical models referring to the conceptual and empirical levels and the important elaboration and description of their combination for scientific practice.

**Conclusion:**

With this article, our intention is to stimulate discussions and reflections on current methods that permeate scientific practice and encourage the use of Theoretical Models as a basis for scientific research in its elaboration, development, and reporting process.

## RESUMO

**Objetivo:**

Apresentar reflexões teórico-metodológicas sobre elaboraçõo, tipos e funções dos modelos teóricos, bem como suas estruturas conceituais e de análise.

**Métodos:**

Trata-se de um ensaio cujo processo de coleta do material foi realizado de forma não sistemática, eleito exclusivamente e baseado na linha de argumentaçõo e reflexão que os autores pretendem submeter à apreciaçõo e ao debate público.

**Resultados:**

Este trabalho apresenta reflexões sobre tipos e funções dos modelos teóricos, fundamentaçõo teórica nas pesquisas e reflexões sobre a importância dos modelos teóricos para as pesquisas em saúde pública e sua relaçõo com o processo de elaboraçõo, desenvolvimento e relato nos estudos científicos. Além disso, são descritos tipos de modelos teóricos referentes aos planos conceitual e empírico, e a importante elaboraçõo e descriçõo da junçõo de ambos para o fazer científico.

**Conclusão:**

Espera-se que este artigo levante discussões e reflexões sobre os métodos atuais que permeiam o fazer científico e fomentem a utilizaçõo dos modelos teóricos como base das pesquisas científicas em seu processo de elaboraçõo, desenvolvimento e relato.

Theoretical models can be characterized as the hypothetical-deductive representation of life or part of it, presenting, as purposes, to know, explain, and/or predict relations at a given moment in time, person, and place^1^. Thus, theoretical models should not be understood only as references or variables cited or introduced in a graphic or analytic chart in a simple way; they are often deemed as a single representation of the empirical level of analysis and/or related to specific sections of a topic^2^. Theoretical models must present the relations established from the theoretical level and the connections between this level and the empirical level, in the form of textual and graphic interpretation and representations.

Causal theoretical models, such as Directed Acyclic Graphs (DAGs), have shown increasing visibility and utility in epidemiological studies^3^. DAGs display assumptions about the relations between variables, often referred to as “nodes,” in the context of graphs. Its form of conceptual theoretical representation intends to minimize biases such as confounding, collision, among others^4,5^. However, although it has promising potential for use in research, the process of preparing and selecting variables is still an important challenge, which requires understanding and theoretical and methodological reflection on the process of creating research variables and understanding, on the part of researchers, of the theoretical and empirical levels for interpreting the data and variables present in the models.

Thus, such reflections drive the potential for scientific reasoning and quality for the creation of DAGs as well as other causal theoretical model formats focused on Epidemiology in Public Health^6^.

Therefore, researchers should reflect on the interaction between the relations observed at the theoretical level and their operationalization at the empirical and analytical levels^7^. Researchers define how the development and use of the theoretical model will take place in their research, including its internal impact (on the validity, quality, strength, and generalization potential of the study) and external impact (on the application of the findings to the decision-making process, the delimitation of protocols and clinical guidelines, recommendations to the population, and on the formulation of public policies). In addition, it is necessary to reflect on the potential of research to generate impacts (positive or negative) on the adopted theory and/or on the theoretical model initially proposed, making the connection between the levels in a deep, dialogic, and dialectical way, thus enabling innovations in science.

**Figure 1.**
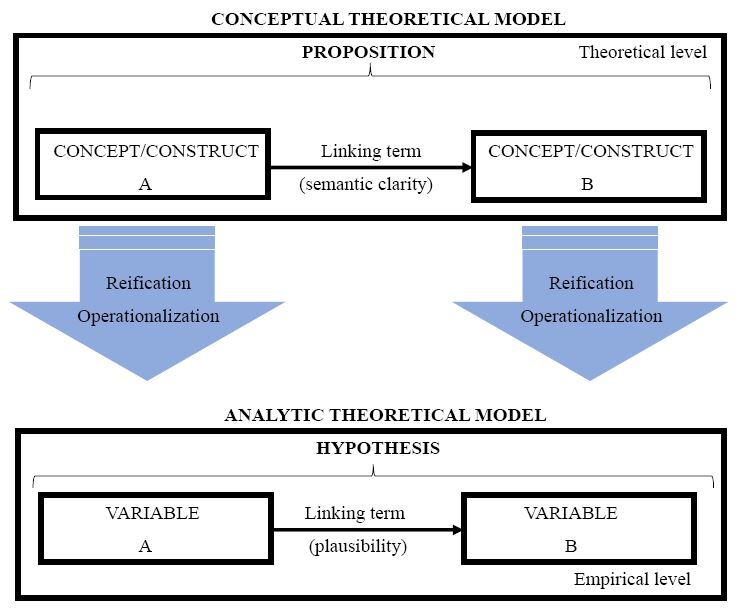
Process of operationalizing concepts in research variables.

Source: prepared by the authors.

It is important for researchers to bear in mind that analytic models are methods that attempt, in a simplified way, to mirror, by their results, the hypothetical-deductive theoretical model that the researcher developed^8^. However, this process is often based only on the reification and operationalization of concepts, that is, the presentation of concepts that can be measurable and are, henceforth, referred to as research variables, as demonstrated in Figure 1.

This process of conceptual transformation for research variables is delicate and, if not well reported, may limit the researchers’ understanding of their complete theoretical reasoning regarding the topic covered in their study^7^. The final process of interpretation and reflection may become fragile, only focused on the results of the analytic model, and deficient when it comes to presenting deep reflections on the initial theoretical-conceptual development and its impact on the results of the study and research in general.

Hence, for research to be interpreted in the best possible way, researchers must present and list sufficient theoretical bases for the readers to appropriate their worldview, considering the time, the person, and the place in which the research originated; that is, the general and idiosyncratic conditions that gave space for the conception, design, operationalization, and reporting of the research. Furthermore, in addition to improving the formal presentation of the research to the external public, favoring transparency in the reported information and facilitating interpretations about the studied relations and the obtained results, the presentation of the theoretical foundation and its representation and adequate reporting allows the research team to reflect on the paths taken during the study and the possibilities of reviewing and improving the theoretical models developed and adopted and/or the outlining of proposals for contributions to existing theories, including those that have already been well-established^9^.

Therefore, it is necessary to reflect that every theoretical model must be understood and explained on the theoretical, empirical, and on both levels, in which each of them are equally important for research and its reports. In addition, we support the idea that all research must present one or more forms of representation of the theoretical models of the study, using the textual format, but also graphic representations, for example, tables, diagrams, graphs, concept maps, among other formats, to systematize and/or categorize concepts and relations.

Choosing a graphic organizer is important when considering the organization and dissemination of knowledge, as it facilitates the understanding of the established relations, the visualization of errors, exaggerations and/or gaps, or excessive simplifications in the theoretical model, favoring the review process, external contributions, and improvement of the presented reasoning^1^. Thus, we support the presentation of theoretical models by a theoretical and conceptual framework, which must be represented and explained visually, preferably with the theoretical one in a textual format and the conceptual one in a graphic format.

Moreover, as aforementioned, we understand that it is necessary to explain all the theoretical models developed in the study, which we characterized into three, considering the theoretical and empirical levels necessary for the development and reporting of scientific research. They are as follows:

- Conceptual Theoretical Model (CTM): understood on the theoretical level, it consists of a propositional network that must present a linking term (preferably textual, with semantic clarity) to express the relation between two or more concepts/constructs;
- Analytic Theoretical Model (ATM): understood on the empirical level, it consists of a network of hypotheses that presents two or more variables connected by a linking term (preferably textual, with the respective scientific plausibility) that indicates the relation between them;
- Observed Theoretical Model (OTM): it is the model resulting from the critical-reflective interpretation of the results observed by the ATM analyses and the initial formulation of the CTM in relation to the research question. The OTM is the connection between the theoretical and the empirical levels, by the researcher’s scientific reasoning. We consider this connection to be extremely important for deeply understanding the potential of the theoretical framework employed and of the influences related to time, person, and place in research.

Thus, we defend as an important aspect for the quality of research the textual and graphic explanation of the theoretical models related to all levels: CTM, ATM, and their connection, the OTM.

During the process of developing the studies and reporting them in the form of a scientific publication, the CTM is initially developed and comprises the combination of the researcher’s previous experiences, hypotheses, findings, and inferences present in the literature^1^. However, the propositions and relations of the CTM consist of concepts and/or constructs that, often, cannot be reified and operationalized to compose variables of the ATM. Subsequently, this may result in the negligence of concepts and/or relations in the face of difficulties in making the concepts reified and operationalized in variables, and/or the operational viability for obtaining, collecting, and/or interpreting them on an empirical level, which may result in simplifications and substantial reductions in the ATM.

As a consequence, there may also be negative impacts on interpretations related to the results observed by the ATM, limiting the scope and depth of the OTM. This process entails the mechanistic and purely operational development of the theoretical model, which basically corresponds to the strict (and restricted) interpretation of the empirical level or of the researcher’s possibilities to operationalize concepts and variables on the empirical level^1^. In this sense, the general conditions for the conception, development, and publicity of research represent different scenarios for outlining the research universe, its scope, and transcendence. Thus, individual characteristics related, for example, to the degree, professional experience, funding availability, and institutional and logistical support, among other issues, may represent different and unequal potential between researchers and research groups.

In the meantime, the categories here expressed as Time, Person, and Place mark the particularities of each research development scenario, whether related to the Time the study was carried out — influencing the support or abandonment of paradigms, theoretical-methodological trends, theoretical-scientific conceptions and developments, recent events or emergencies that require special attention and end up defining research priorities, exalting certain topics at the expense of others that may be obfuscated, etc.; to the Person — in the sense of idiosyncrasies and previous experiences of the researcher and the research team, the possibility of external influences, and the scientific relevance of the social circle of influence of the researcher, etc.; and to the Place — in which the developments take place, with certain trends or orientations being supported or discouraged because of demographic, epidemiological, cultural, and/or social specificities.

For decades, the lack of reflection on the use of theoretical models and their variables have been entailing erroneous results regarding a given topic and leading to premature conclusions and to the lack of reliability and reproducibility of research. For instance, we can mention the case of weather forecasting, in which mathematical models are used to forecast the weather around the world, but the lack of consideration of important variables, such as cloud formation and heat distribution, can lead to inaccurate forecasts^10^.

Furthermore, theoretical models are often used to explain the relation between risk factors and diseases, but the lack of consideration of all relevant variables may lead to inadequate conclusions. For example, authors of a study published in 2011 concluded that the use of vitamin E supplements increased the risk of prostate cancer in men^11^. Nevertheless, the study did not consider the dosage or duration of use of the supplements, which considerably influences the results and may have led to an erroneous conclusion on the part of the research team. Thus, it is essential that researchers critically reflect on how to use and develop theoretical models in their research.

In this sense, the explanation — textual and graphic — of the theoretical path followed in the research, through the theoretical and empirical levels by the CTM, ATM, and OTM, allows a deeper understanding of the research conditions and their particularities. For more details about the process of elaboration, use, and reporting of theoretical models in epidemiological research, we recommend to access the available references^1,7,9^.

**Figure 2.**
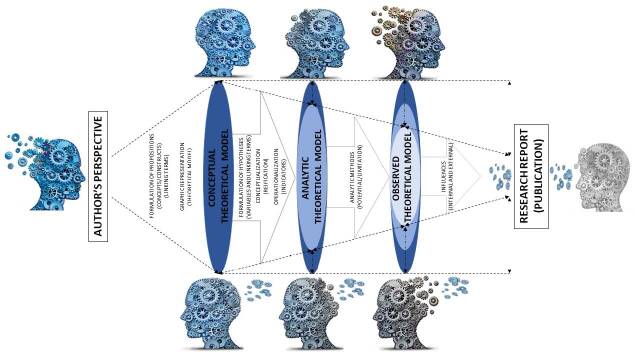
Representation of the theoretical-methodological path followed in research, going through the theoretical and empirical levels by the Conceptual, Analytic, and Observed theoretical models.

Source: prepared by the authors.

In addition, explanations favor the understanding of the authors’ line of reasoning; of the potential of the study and its limitations in theoretical and methodological terms; and a better interpretation of the research, favoring a deep reflection on the expected, possible, and observed relations, which may support contributions to the CTM initially proposed and establish a feedback process for the adopted theoretical foundation, which may favor innovations for the theoretical foundation and for science altogether, as demonstrated in Figure 2.

Hence, understanding the thinking and the theoretical-methodological path taken by the primary researcher becomes indispensable when thinking about the transposition and expansion of knowledge as well as for the reproducibility of research.

In addition, it should be noted that although we are addressing reproducibility and the possible objectivity of the studies, we must understand that all scientific research presents important marks of subjectivity, which differentiates and/or highlights them from the others, and which may influence their acceptance or rejection, considering the relations circumscribed in a given time and space, and the characteristics related to the person(s).

All in all, with this manuscript, we sought to promote and strengthen not scientific reproducibility itself, but the understanding of human identity and subjectivity behind scientific practice, and the necessary reflections and explanations of these singularities as a path of transparency and quality for scientific reporting, a condition without which reproducibility may be unfeasible. In other words, in order to accept and consider reproducibility as an important aspect of the scientific practice, it is essential that the studies presented to the scientific community promote real conditions for reproducibility to occur.

To this end, the complete and in-depth report of studies regarding the theoretical foundation that underlies them, the theoretical path taken, the methodological considerations and choices, the methods for interpreting the results and limitations, and the insights deriving from this process, become an indispensable condition.
